# The relationship between the composite dietary antioxidant index and rheumatoid arthritis risk in American adults: the mediating role of BMI

**DOI:** 10.3389/fimmu.2025.1560570

**Published:** 2025-04-09

**Authors:** Tao Gao, Zhi-Yu Chen, Tao Li, Jian-Dong Tang, Xu Lin, Hai-Gang Hu, Sheng-Yu Wan, Chao Wu

**Affiliations:** ^1^ Orthopaedics of Zigong Fourth People’s Hospital, Zigong, Sichuan, China; ^2^ Respiratory Medicine of Zigong Fourth People’s Hospital, Zigong, Sichuan, China

**Keywords:** rheumatoid arthritis, composite dietary antioxidant index, body mass index, mediating role, NHANES

## Abstract

**Background:**

The Clinical Dietary Antioxidant Index (CDAI) is a dietary antioxidant assessment index. Although CDAI has been shown to play a role in various chronic diseases, its association with rheumatoid arthritis (RA) remains underexplored. The aim of this study was to investigate the relationship between the CDAI and RA in U.S. adults, and to examine the mediating role of body mass index (BMI) in the relationship between CDAI and RA incidence.

**Methods:**

This cross-sectional study utilized data from the 2015–2023 National Health and Nutrition Examination Survey (NHANES). Weighted multivariate logistic regression models, restricted cubic spline (RCS) functions, and subgroup analyses were employed to examine the association between CDAI levels and RA in American adults. Mediation analysis was conducted to explore the mediating role of BMI in the relationship between CDAI and RA incidence.

**Results:**

A total of 13,288 participants were included, of whom 787 were diagnosed with RA, with a prevalence rate of 5.9%. Weighted multivariate logistic regression analyses across all four models showed a negative correlation between CDAI levels in the highest quartile and RA incidence. Restricted cubic spline curves revealed a non-linear negative association between CDAI, vitamin E, carotenoids, selenium, and RA incidence. As levels of CDAI, vitamin E, carotenoids, and selenium increased, the risk of RA decreased. Subgroup analyses and forest plots indicated significant associations between CDAI levels and RA across subgroups, including females, individuals aged over 60 years, Other Race - Including Multi-Racial groups, smokers, non-drinkers, individuals with heavy physical activity, hypertension, and BMI >25 (P < 0.05). Mediation analysis showed that BMI partially mediated the relationship between CDAI and RA, accounting for 10.88% of the effect.

**Conclusions:**

CDAI levels were negatively associated with RA incidence, particularly the components of CDAI such as vitamin E, carotenoids, and selenium. BMI mediated the relationship between CDAI and RA.

## 1 Introduction

Rheumatoid arthritis (RA) is a common chronic autoimmune disease primarily characterized by inflammation, swelling, and pain in multiple joints, which can lead to joint destruction and functional impairment over time (1). A systematic review revealed that RA incidence varies across countries, with the highest reported in Cuba (2.7%) and the lowest in the Philippines (0.17%) (2). Additionally, a Global Burden of Disease study indicated that the global burden of RA has been increasing steadily over the past 30 years, with the number of RA patients projected to rise from approximately 1.07 million at the end of 2019 to about 1.5 million by 2040 (3). Given the rising global burden of RA, identifying effective preventive strategies is crucial for public health. The increasing prevalence of RA underscores the need for early intervention and lifestyle modifications to reduce disease risk.

RA patients often experience significant chronic inflammation and oxidative stress, which not only exacerbate disease progression but also substantially reduce their quality of life (4, 5). Although current treatment options focus on immunosuppressive and anti-inflammatory drugs (6, 7), they have not fully addressed the underlying mechanisms of RA onset and progression. In recent years, dietary interventions have gained widespread attention as a potential strategy for the prevention and management of RA. Hulander et al. (8) demonstrated that an anti-inflammatory diet—characterized by high intake of fatty fish, whole grains, fruits, nuts, and berries—can reduce systemic inflammation and lower ESR levels in RA patients. Another study by Hulander et al. (9) showed that an anti-inflammatory diet improves lipid profiles in RA patients, offering cardiovascular protection.

The Composite Dietary Antioxidant Index (CDAI) is a comprehensive metric used to evaluate dietary antioxidant levels. It integrates the intake of multiple antioxidants, including vitamins A, C, and E, carotenoids, zinc, and selenium, and reflects the overall capacity of dietary patterns to counteract inflammation and oxidative stress. Previous studies have shown that CDAI is negatively associated with the risk of several chronic diseases, such as diabetes, hypertension, and fatty liver (10–12). CDAI is a comprehensive measure of dietary antioxidants, such as vitamin E, carotenoids, and selenium, which have been shown to play a significant role in inflammation and oxidative stress (13–16)—key factors in the pathogenesis of RA. While the Dietary Inflammatory Index and the Healthy Eating Index are also relevant, CDAI offers a more comprehensive antioxidant index that specifically relates to reducing oxidative stress, a hallmark of RA. However, the potential association between CDAI and RA risk has not been extensively validated, especially in large, population-based studies.

This study aims to explore the association between CDAI and RA risk in American adults using data from the 2015–2023 National Health and Nutrition Examination Survey (NHANES) and further evaluate the mediating role of body mass index (BMI) in this relationship. We hypothesize that higher dietary antioxidant intake (reflected by higher CDAI) is associated with a reduced risk of RA and that BMI partially mediates this relationship. In addition, we consider BMI as a mediator rather than a confounder. BMI plays a key role in modulating inflammation, as adipose tissue releases pro-inflammatory cytokines (e.g., IL-6, TNF-α) (17), which increase the inflammatory burden. Given that oxidative stress and inflammation are critical mechanisms in the pathogenesis of RA, we hypothesize that BMI may act as a mediator in the relationship between CDAI and RA risk.

## 2 Participants and methods

### 2.1 Study population

This study is a cross-sectional analysis utilizing data from the 2015–2023 National Health and Nutrition Examination Survey (NHANES), a nationally representative health and nutrition survey targeting non-institutionalized adults in the United States. The detailed survey methodology and data collection processes for NHANES have been approved by the National Center for Health Statistics (NCHS) Review Board and are publicly available on its official website. All participants provided informed consent.

**Figure 1:**
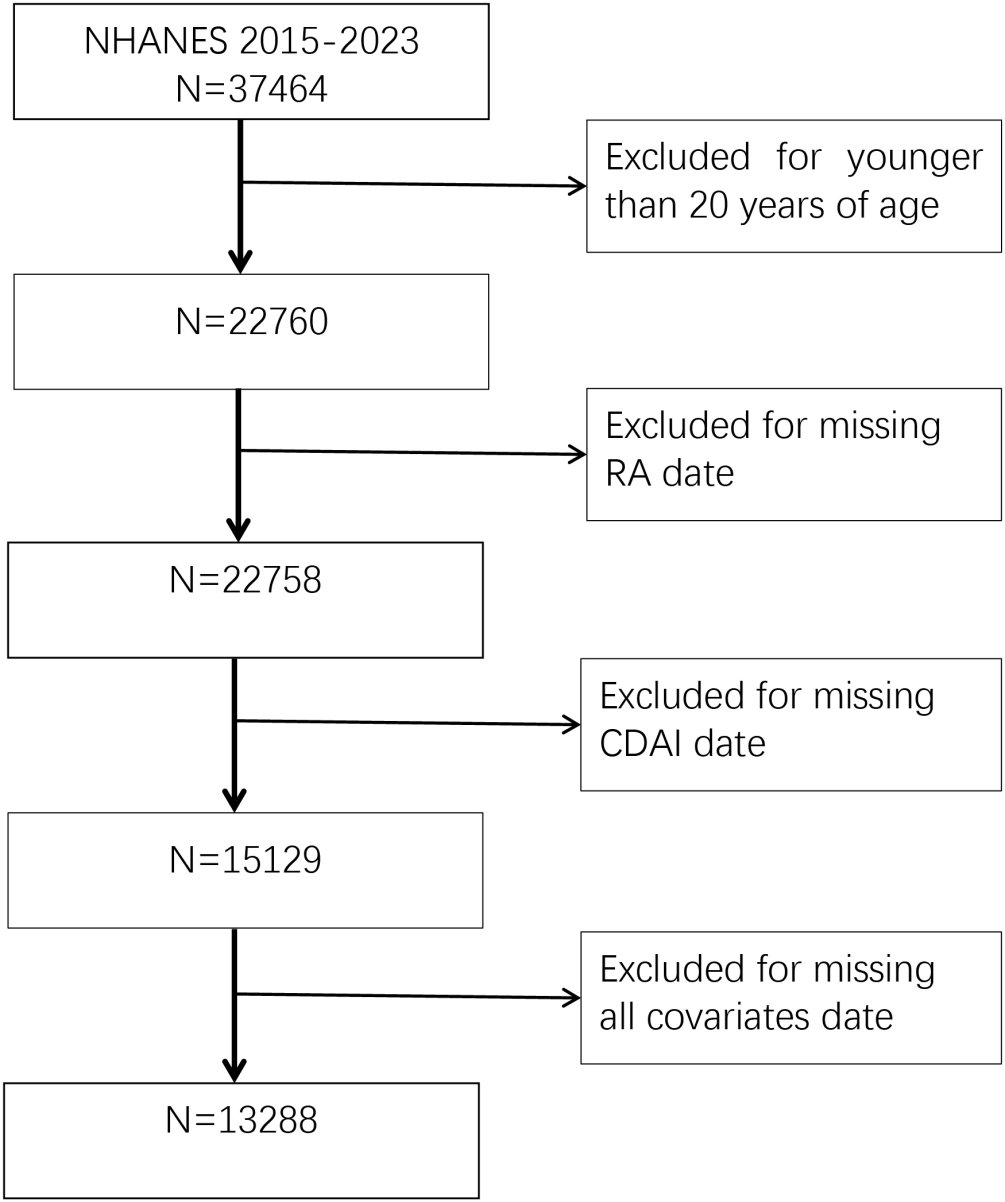
Flowchart of sample selection.

This study includes data from 37,464 individuals who participated in the NHANES survey between 2015 and 2023. We excluded participants under the age of 20, as well as those with missing data on rheumatoid arthritis (RA), CDAI, BMI, HSCRP, SII, physical activity, hypertension, smoking, alcohol consumption, and other covariates. After exclusions, a total of 13,288 participants were included in the analysis (**Figure 1**).

### 2.2 Exposure variables: CDAI assessment

The Composite Dietary Antioxidant Index (CDAI) was calculated using 24-hour dietary recall data from the NHANES database. We referenced the modified CDAI calculation method proposed by Wright et al. (18), which involves the intake of six dietary antioxidants (vitamins A, C, E, carotenoids, zinc, and selenium). Specifically, the intake of each antioxidant was standardized (i.e., by subtracting the mean and dividing by the standard deviation), and then the standardized intake values of the antioxidants were summed to obtain the CDAI score. Higher CDAI scores indicate higher intake of dietary antioxidants.

### 2.3 Outcome variables: RA assessment

The diagnosis of RA was based on the medical conditions questionnaire in NHANES, with the question: “Has a doctor or other health professional ever told you that you have arthritis?” If the answer was “Yes,” participants proceeded to the next question: “What type of arthritis?” Participants who answered “Rheumatoid Arthritis” were classified into the RA group, while the rest were categorized as non-RA.

### 2.4 Covariates determination

We considered potential covariates that could influence the relationship between CDAI and RA, including gender, age, race, BMI, smoking status, alcohol consumption, hypertension, physical activity, serum HSCRP levels, and SII. Data for gender, age, race, smoking, alcohol consumption, and physical activity were collected via questionnaires. Race was categorized into the following groups: Mexican American, Other Hispanic, Non-Hispanic White, Non-Hispanic Black, and Other Race - Including Multi-Racial. Smoking status was classified based on questionnaire responses into “Yes” or “No.” Alcohol consumption was categorized based on the question: “During the past 12 months, on those days that you drank alcoholic beverages, on the average, how many drinks did you have?” The results were classified as heavy (men: ≥3 drinks/day or women: ≥2 drinks/day), moderate (men: 1–2 drinks/day or women: 1 drink/day), or none (19). Hypertension was defined based on the average of three measurements of systolic and diastolic blood pressure, with hypertension defined as systolic blood pressure ≥130 mmHg or diastolic blood pressure ≥80 mmHg (20). Physical activity was categorized as Heavy, Moderate, or Insufficient based on responses to questions about vigorous recreational or work activities. Participants who reported vigorous activity in either category were classified as “Heavy,” moderate activity in either category was classified as “Moderate,” and the remaining participants were classified as “Insufficient.” BMI was assessed based on physical examination results and categorized into three groups: <25, 25–30, and >30. Serum HSCRP levels were measured using laboratory test results. SII was calculated from blood analysis using neutrophil, lymphocyte, and platelet counts, as follows: SII = (platelet count × neutrophil count)/lymphocyte count.

### 2.5 Statistical analysis

The NHANES data were weighted using the survey weights provided by the National Center for Health Statistics (NCHS) to ensure the sample is representative of the U.S. adult population. Continuous variables that followed a normal distribution were expressed as weighted means ± standard deviation (SD), while categorical variables were expressed as weighted percentages. Differences were analyzed using weighted Student’s t-test and weighted χ² test.

To evaluate the relationship between CDAI and RA, weighted multivariate logistic regression models were used. Four models were constructed to adjust for covariates:

Model 1: No covariate adjustment.
Model 2: Adjusted for gender, age, and race.
Model 3: Adjusted for gender, age, race, smoking, alcohol consumption, hypertension, physical activity, HSCRP, and SII.
Model 4: Adjusted for all variables in Model 3 + BMI.

We used restricted cubic splines (RCS) to assess the non-linear relationship between CDAI and RA. Subgroup analyses and forest plots were used to further evaluate the relationship between CDAI and RA in various subgroups, The subgroups were selected based on known risk factors for RA, including age, gender, BMI, and smoking status. Mediation analysis was performed to assess the mediating role of BMI in the relationship between CDAI and RA, and the mediation effect proportion was calculated.

Statistical analyses were conducted using R software (version 4.2.0). A P-value <0.05 was considered statistically significant.

## 3 Results

### 3.1 Participant characteristics

A total of 13,288 participants were included in this study, of whom 787 were diagnosed with rheumatoid arthritis (RA) (5.9%) and 12,501 were non-RA participants (94.1%). Statistically significant differences (P < 0.05) were observed between the two groups in terms of age, race, BMI, smoking status, drinking status, activity status, hypertension status, HSCRP, vitamin E, carotene, zinc, selenium, SII, and CDAI (**Table 1**).

**Table 1:** Characteristics of included samples by RA.

Variables	Total (n = 13288)	Non-RA (n = 12501)	RA (n = 787)	p
Gender (%)				0.463
Male	6245 (48.70)	5887 (48.62)	358 (50.66)	
Female	7043 (51.30)	6614 (51.38)	429 (49.34)	
Age (%)				<0.001
20-40	3741 (35.79)	3693 (36.77)	48 (12.08)	
41-60	4242 (34.82)	3999 (34.71)	243 (37.51)	
>60	5305 (29.39)	4809 (28.52)	496 (50.41)	
Race (%)				0.000
Mexican American	1508 (8.03)	1418 (8.01)	90 (8.54)	
Other Hispanic	1432 (7.88)	1324 (7.82)	108 (9.39)	
Non-Hispanic White	5924 (63.76)	5643 (64.05)	281 (56.58)	
Non-Hispanic Black	2686 (10.51)	2449 (10.23)	237 (17.10)	
Other Race - Including Multi-Racial	1738 (9.83)	1667 (9.89)	71 (8.39)	
BMI (%)				0.001
<25	2564 (19.77)	2472 (20.04)	92 (13.15)	
25-30	4995 (39.17)	4711 (39.30)	284 (36.22)	
>30	5729 (41.06)	5318 (40.66)	411 (50.64)	
Smoke status (%)				<0.001
Yes	5566 (40.55)	5144 (39.97)	422 (54.62)	
No	7722 (59.45)	7357 (60.03)	365 (45.38)	
Drinking status (%)				0.005
Heavy	4446 (37.16)	4227 (37.42)	219 (30.71)	
Moderate	4687 (36.35)	4443 (36.39)	244 (35.35)	
None	4155 (26.49)	3831 (26.18)	324 (33.94)	
Activity status (%)				<0.001
Heavy	5681 (48.72)	5417 (49.26)	264 (35.66)	
Moderate	4134 (31.08)	3867 (30.90)	267 (35.48)	
Insufficient	3471 (20.20)	3215 (19.84)	256 (28.85)	
Hypertension status (%)				0.000
Yes	5541 (35.60)	5138 (35.15)	403 (46.50)	
No	7747 (64.40)	7363 (64.85)	384 (53.50)	
HSCRP (mean (SD))	3.819 (7.114)	3.742 (6.987)	5.683 (9.509)	0.001
Vitamin E (mean (SD))	10.182 (8.003)	10.216 (8.011)	9.361 (7.748)	0.025
Vitamin A (mean (SD))	1015.673 (853.360)	1008.429 (758.202)	1191.081 (2101.162)	0.146
Carotene (mean (SD))	9076.804 (9017.642)	9135.790 (9081.511)	7648.477 (7160.474)	0.000
Vitamin C (mean (SD))	76.276 (70.088)	76.318 (70.212)	75.261 (67.049)	0.718
Zinc (mean (SD))	10.531 (5.238)	10.558 (5.263)	9.870 (4.553)	0.017
Selenium (mean (SD))	111.439 (52.203)	111.794 (52.443)	102.838 (45.173)	0.003
SII (mean (SD))	535.218 (315.352)	532.657 (308.313)	597.224 (449.439)	0.005
CDAI Quartiles (%)				0.033
1	3311 (22.47)	3068 (22.21)	243 (28.72)	
2	3311 (24.73)	3120 (24.78)	191 (23.61)	
3	3326 (25.94)	3129 (25.92)	197 (26.43)	
4	3340 (26.87)	3184 (27.10)	156 (21.24)	

Categorical variables are expressed as unweighted counts (weighted percentages); continuous data are expressed as weighted means (SD). BMI, body mass index; SII, systemic inflammatory index; CDAI, Composite Dietary Antioxidant Index.

### 3.2 CDAI and RA correlation analysis

Weighted multivariate logistic regression analyses across all four models showed a negative correlation between CDAI levels in the highest quartile and RA incidence. Using the lowest quartile as the reference, the unadjusted model revealed an odds ratio (OR) of 0.61 (95% CI: 0.43–0.85, P = 0.004) for the highest CDAI quartile. The model adjusted for gender, race, and age showed an OR of 0.62 (95% CI: 0.45–0.87, P = 0.006). The model adjusted for all variables except BMI showed an OR of 0.70 (95% CI: 0.50–0.99, P = 0.043), and the fully adjusted model, including BMI, showed an OR of 0.71 (95% CI: 0.51–1.00, P = 0.048). Furthermore, in the unadjusted model and the model adjusted for gender, race, and age, vitamin E and carotene, as well as selenium in the unadjusted model, also demonstrated a negative correlation with RA incidence in the highest quartile (P < 0.05) (**Table 2**).

**Table 2:** The correlation between CDAI and RA by multivariable logistic regression.

Variables	Model1		Model2		Model3		Model4	
	OR (95%CI)	P	OR (95%CI)	P	OR (95%CI)	P	OR (95%CI)	P
CDAI Quartiles								
1	1.00 (Reference)		1.00 (Reference)		1.00 (Reference)		1.00 (Reference)	
2	0.74 (0.53, 1.03)	0.076	0.75 (0.54, 1.05)	0.089	0.81 (0.57, 1.13)	0.205	0.81 (0.58, 1.13)	0.212
3	0.79 (0.56, 1.10)	0.16	0.83 (0.59, 1.16)	0.268	0.91 (0.63, 1.30)	0.585	0.91 (0.64, 1.30)	0.594
4	0.61 (0.43, 0.85)	0.004	0.62 (0.45, 0.87)	0.006	0.7 (0.50, 0.99)	0.043	0.71 (0.51, 1.00)	0.048
Vitamin E Quartiles								
1	1.00 (Reference)		1.00 (Reference)		1.00 (Reference)		1.00 (Reference)	
2	0.83 (0.60, 1.13)	0.232	0.85 (0.62, 1.16)	0.301	0.91 (0.66, 1.27)	0.588	0.91 (0.65, 1.26)	0.559
3	0.76 (0.53, 1.08)	0.126	0.81 (0.56, 1.16)	0.236	0.91 (0.62, 1.33)	0.602	0.9 (0.62, 1.32)	0.591
4	0.67 (0.49, 0.91)	0.011	0.73 (0.54, 0.99)	0.045	0.83 (0.60, 1.15)	0.251	0.84 (0.61, 1.15)	0.27
Carotene Quartiles								
1	1.00 (Reference)		1.00 (Reference)		1.00 (Reference)		1.00 (Reference)	
2	0.86 (0.63, 1.16)	0.309	0.88 (0.65, 1.19)	0.406	0.94 (0.70, 1.27)	0.69	0.94 (0.70, 1.27)	0.69
3	0.84 (0.59, 1.21)	0.35	0.89 (0.62, 1.27)	0.508	0.97 (0.68, 1.39)	0.863	0.98 (0.68, 1.42)	0.927
4	0.66 (0.48, 0.91)	0.011	0.67 (0.48, 0.92)	0.016	0.74 (0.54, 1.02)	0.069	0.75 (0.54, 1.04)	0.084
Zinc Quartiles								
1	1.00 (Reference)		1.00 (Reference)		1.00 (Reference)		1.00 (Reference)	
2	0.96 (0.73, 1.27)	0.784	1.01 (0.76, 1.34)	0.924	1.06 (0.79, 1.42)	0.676	1.06 (0.79, 1.42)	0.671
3	0.88 (0.66, 1.17)	0.373	0.96 (0.72, 1.28)	0.781	1.03 (0.77, 1.39)	0.836	1.04 (0.77, 1.39)	0.807
4	0.78 (0.57, 1.08)	0.138	0.87 (0.63, 1.20)	0.377	0.92 (0.67, 1.29)	0.633	0.92 (0.66, 1.28)	0.607
Selenium Quartiles								
1	1.00 (Reference)		1.00 (Reference)		1.00 (Reference)		1.00 (Reference)	
2	0.85 (0.61, 1.19)	0.336	0.87 (0.62, 1.21)	0.401	0.9 (0.64, 1.26)	0.531	0.9 (0.64, 1.26)	0.529
3	0.67 (0.51, 0.89)	0.007	0.71 (0.52, 0.96)	0.026	0.74 (0.54, 1.01)	0.059	0.73 (0.54, 1.00)	0.047
4	0.68 (0.47, 0.98)	0.038	0.75 (0.52, 1.09)	0.124	0.79 (0.54, 1.15)	0.204	0.78 (0.53, 1.14)	0.189

OR, Odds Ratio; CI, Confidence Interval.

Model1: Crude.

Model2: Adjust: Gender, Race, age_custom.

Model3: Adjust: Gender, Race, HSCRP, Smoke_status, Drinking_status, Activity_status, Hypertension_status, SII, age_custom.

Model4: Adjust: Gender, Race, HSCRP, Smoke_status, Drinking_status, Activity_status, Hypertension_status, SII, age_custom, BMI.

**Figure 2:**
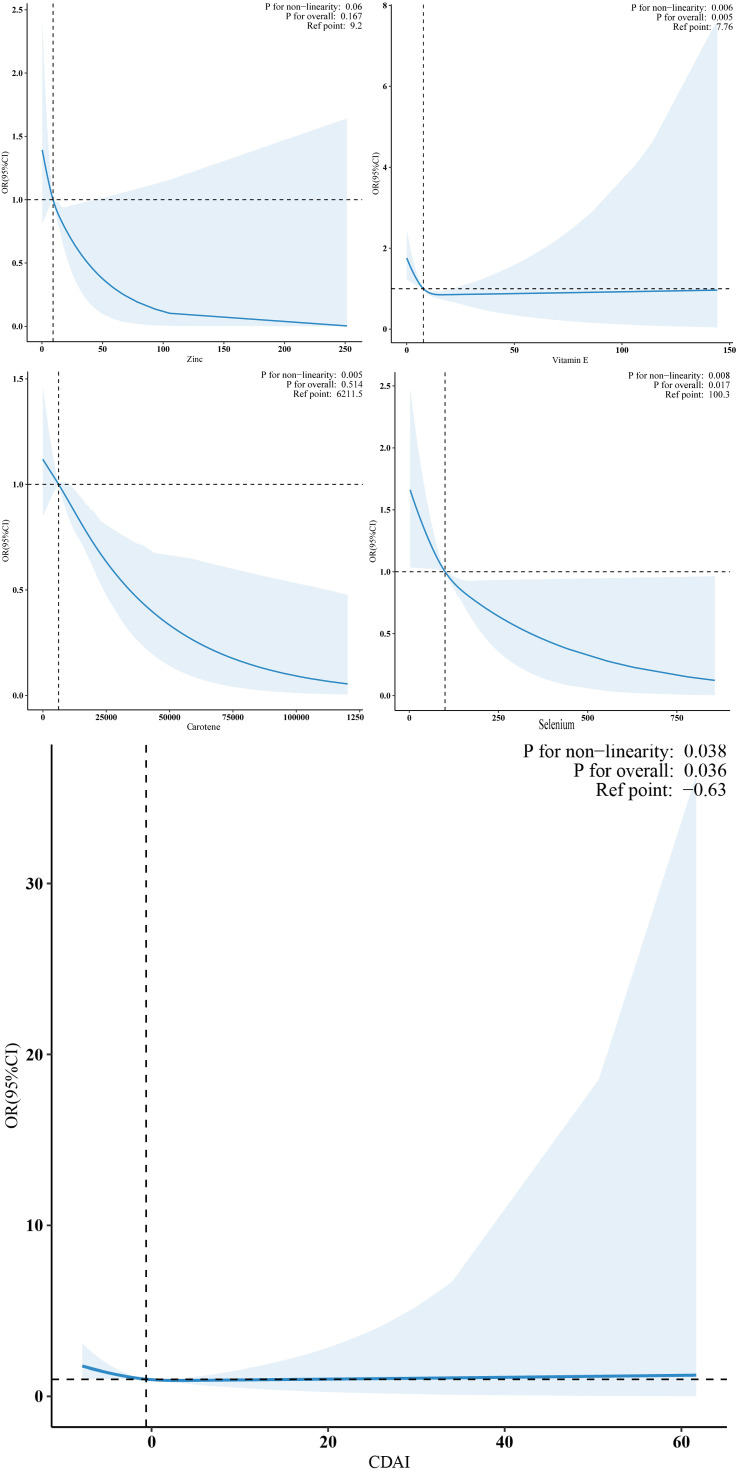
Restricted cubic spline curve (RCS) plot of the relationship between CDAI, vitamin E, carotene, selenium, and RA incidence.

Restricted cubic spline (RCS) analysis showed a non-linear negative correlation between CDAI, vitamin E, carotene, selenium, and RA incidence. As CDAI, vitamin E, carotene, and selenium levels increased, the risk of RA decreased (P < 0.05) (**Figure 2**).

### 3.3 Subgroup analysis of CDAI and RA

**Figure 3:**
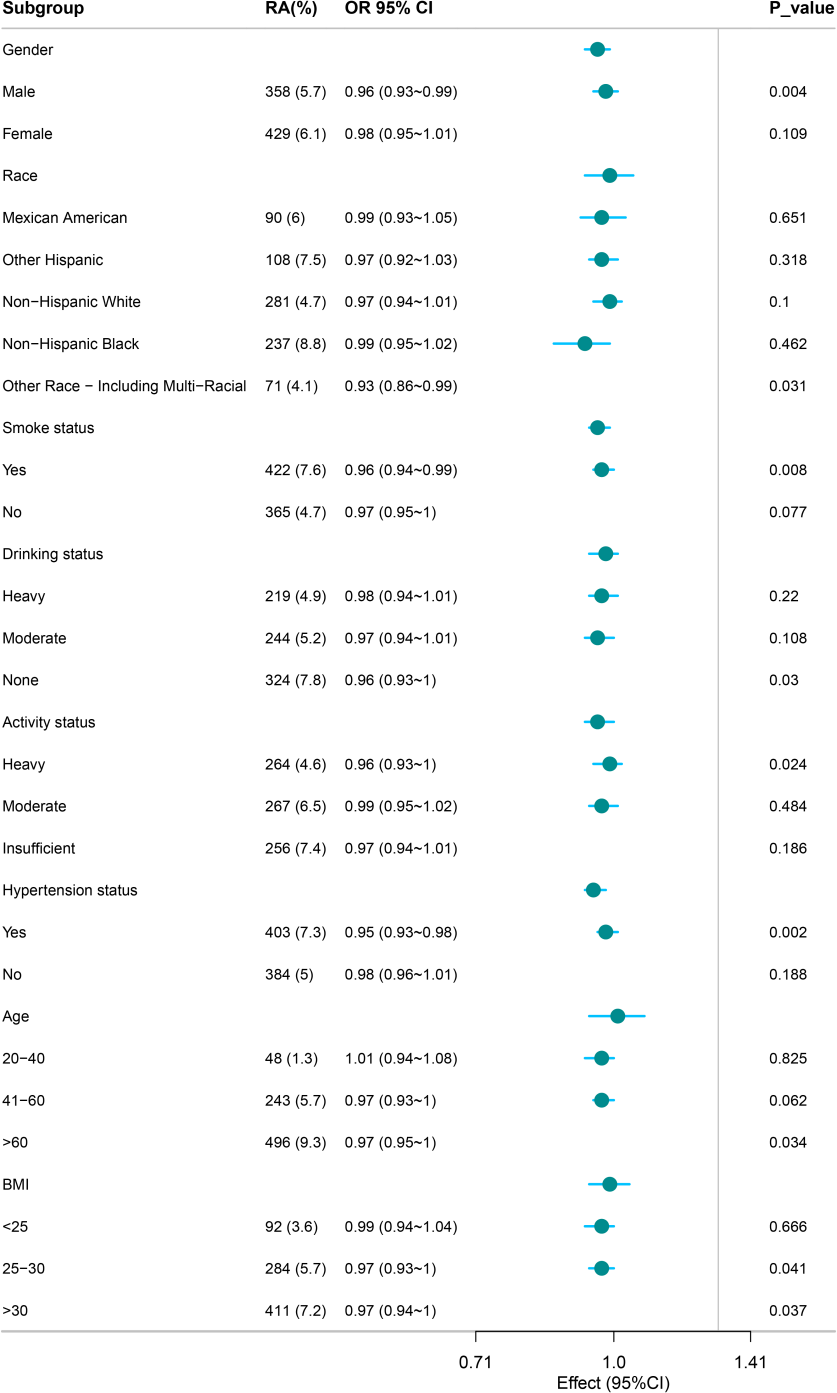
Subgroup analysis of the relationship between CDAI and RA.

Subgroup analysis revealed that CDAI was negatively correlated with RA in males, individuals of “Other Race - Including Multi-Racial” ethnicity, smokers, non-drinkers, individuals with heavy activity status, those with hypertension, individuals aged >60, and those with BMI >25 (P < 0.05) (**Figure 3**).

### 3.4 Causal mediation analysis

**Figure 4:**
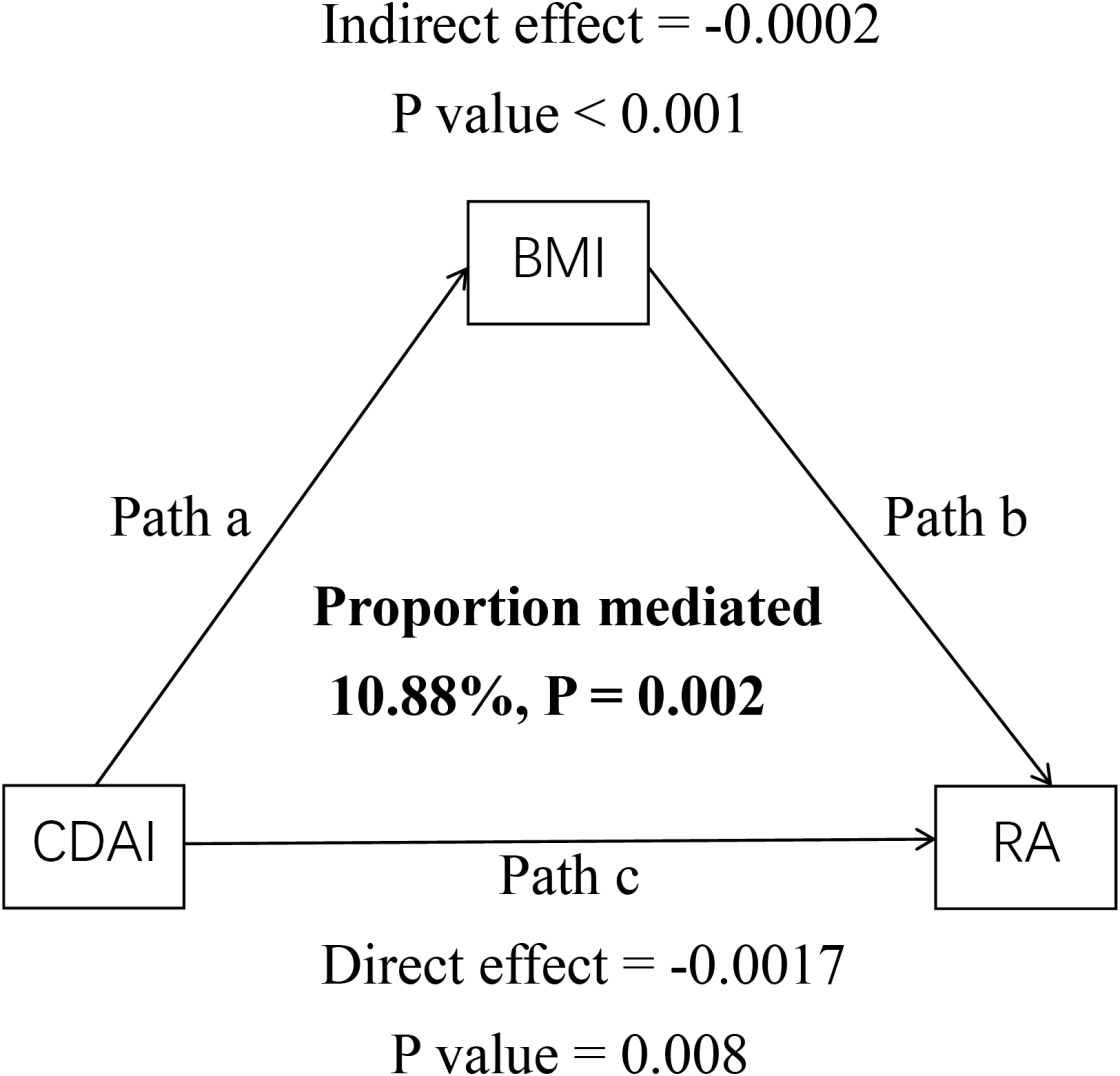
Mediated analysis model path diagram. Notes: CDAI was defined as the independent variable; RA as the dependent variable; and BMI as the mediating variable. Path a represents the regression coefficient of the association between CDAI and BMI. Path b represents the regression coefficient of the association between BMI and RA. Path c represents the direct effect of CDAI on RA.

We performed causal mediation analysis to further investigate the potential mediating role of BMI in the relationship between CDAI and RA. The mediation model and its paths are shown in **Figure 4**, where CDAI is the independent variable, BMI is the mediator, and RA is the dependent variable. The results showed that CDAI indirectly influenced the occurrence of RA through BMI, with an indirect effect of -0.0002 (95% CI: -0.0003, -0.0001, P < 0.001). This indicates that BMI partially mediated the relationship between CDAI and RA, accounting for 10.88% of the effect (**Table 3**).

**Table 3:** Mediation analysis of BMI in the association between CDAI and RA.

Effect Decomposition	Coefficient (95% CI)	P value	Proportion mediated, %
Indirect Effect	-0.0002 (-0.0003,-0.0001)	0.008	10.88%
Direct Effect	-0.0017 (-0.0028, -0.0006)	0.002	89.12%
Total Effect	-0.0019 (-0.003, -0.0008)	0.002	

## 4 Discussion

### 4.1 Negative correlation between CDAI and RA

In this study, we found a significant negative correlation between the Composite Dietary Antioxidant Index (CDAI) and the risk of rheumatoid arthritis (RA), revealing the important role of dietary antioxidants in the pathophysiology of RA. Specifically, higher CDAI (i.e., higher antioxidant intake) was associated with a lower risk of RA, particularly at the highest quartile level, where the relative risk of RA was significantly reduced. This negative correlation reflects the potential protective effects of dietary antioxidants against RA, and the following mechanisms may explain this association:

#### 4.1.1 Oxidative stress and the pathogenesis of RA

Oxidative stress is considered one of the core mechanisms underlying RA development (21, 22). Altered antioxidant system and elevated serum and synovial fluid lipid peroxidation levels in patients with RA (23).The levels of oxidative stress in RA patients are significantly elevated, primarily due to the excessive production of reactive oxygen species (ROS) and reactive nitrogen species (RNS) (24, 25). These free radicals attack the synovial membrane, cartilage, and bone tissue, inducing inflammatory responses and exacerbating joint damage (25). In addition, ROS are able to activate or deactivate different types of receptors, proteins and signaling molecules. This consequently leads to cellular dysfunction, which in turn leads to the development of the pathological condition of rheumatoid arthritis (23). Oxidative stress also activates pro-inflammatory signaling pathways, such as NF-κB and JNK, further increasing the release of pro-inflammatory cytokines (e.g., TNF-α, IL-6, IL-1β), thereby amplifying the inflammatory response (26).

Dietary antioxidants (such as vitamin E, carotenoids, and selenium) can reduce oxidative stress-induced damage to joint tissues by scavenging free radicals and inhibiting the activation of pro-inflammatory signaling pathways (27–29). This explains why higher CDAI levels are associated with a significantly lower risk of RA.

#### 4.1.2 Protective role of vitamin E in RA

Vitamin E, a major component of CDAI, is a potent lipid-soluble antioxidant that effectively scavenges ROS and lipid peroxides, protecting cell membranes and tissues from oxidative damage (14, 30). Kou H et al (31) showed that regular use of vitamin E supplements can help RA patients reduce joint discomfort, edema and stiffness and improve their overall quality of life. Recent results from a large cross-sectional study of 2906 participants suggest a beneficial relationship between increased dietary vitamin E intake and reduced risk of all-cause mortality in patients with RA (32). In addition, vitamin E exerts anti-inflammatory effects through several mechanisms:

**Inhibition of NF-κB signaling**: NF-κB is a key pro-inflammatory transcription factor involved in regulating the expression of various pro-inflammatory cytokines (33). In RA patients, the activation of the NF-κB pathway is closely related to joint inflammation (34). Vitamin E can reduce the release of pro-inflammatory cytokines (such as IL-1β, TNF-α) by inhibiting NF-κB activity (35), thereby alleviating joint inflammation.
**Immune Response Modulation**: Vitamin E enhances the function of T and B cells, promotes the secretion of anti-inflammatory cytokines (e.g., IL-10), and inhibits the expression of Th17 cells and pro-inflammatory cytokines (36, 37). This immunomodulatory effect may help mitigate the immune-mediated inflammatory response in RA.

#### 4.1.3 Anti-inflammatory mechanism of carotenoids

Carotenoids are a class of plant flavonoid compounds with strong antioxidant properties (38). Carotenoids not only directly scavenge ROS but also exert immune-regulatory effects through their metabolites (e.g., retinol). The specific protective mechanisms of carotenoids in RA include:

**Scavenging Free Radicals**: Carotenoids, as effective free radical scavengers, can reduce oxidative stress levels in RA patients, protecting synovial cells and cartilage tissue from oxidative damage (39, 40).
**Regulation of Immune Response**: Carotenoids can inhibit the expression of pro-inflammatory cytokines (such as IL-6, TNF-α) (41) and promote the release of anti-inflammatory cytokines (such as IL-10) (42), thereby exerting anti-inflammatory and immune-modulatory effects in RA patients. Additionally, carotenoids can regulate macrophage function and reduce the spread of inflammatory responses (15).

#### 4.1.4 Anti-inflammatory and immune-regulatory effects of selenium

Selenium, a key trace element in CDAI, exerts its antioxidant effects primarily through its role as a cofactor in glutathione peroxidase (GPx) and selenoprotein P (16). The specific protective mechanisms of selenium in RA include:

**Enhancing Antioxidant Defense**: Selenium, as a core component of GPx, promotes the breakdown of hydrogen peroxide and organic peroxides, reducing oxidative stress-induced damage to synovial cells and cartilage (43, 44). Selenium’s antioxidant role is particularly important for RA patients, as they often have elevated lipid peroxide levels.
**Inhibition of Inflammatory Cytokine Expression**: Selenium can inhibit the expression of pro-inflammatory cytokines by affecting signaling pathways such as NF-κB and Nrf2, thereby reducing the inflammatory response in synovial cells and macrophages (45).
**Immune Function Modulation**: Selenium regulates T and B cell functions, promotes the secretion of anti-inflammatory cytokines, and inhibits the proliferation of Th1 and Th17 cells (46), which in turn suppresses the immune-inflammatory response associated with RA.

#### 4.1.5 Role of CDAI in overall dietary patterns

CDAI comprehensively assesses the intake of various antioxidants in the diet, thus reflecting not only individual nutrient intake but also the overall dietary pattern. A high CDAI score is usually associated with high intake of fruits, vegetables, nuts, and whole grains, all rich in antioxidants and anti-inflammatory compounds that exert a combined anti-inflammatory and immune-modulatory effect.

**Mediterranean Diet Pattern**: The high CDAI score resembles the Mediterranean diet, which is rich in olive oil, fish, fruits, and vegetables. Numerous studies have shown that the Mediterranean diet can reduce the risk of RA and improve symptoms in RA patients (47).
**Role of Dietary Fiber**: High CDAI scores are often associated with higher dietary fiber intake. Dietary fiber can reduce RA risk by altering the gut microbiota and increasing the production of anti-inflammatory short-chain fatty acids (e.g., butyrate) (48), thereby exerting an anti-inflammatory effect.

In conclusion, the negative correlation between CDAI and RA observed in this study may be attributed to the multifaceted protective mechanisms of dietary antioxidants, including scavenging free radicals, inhibiting pro-inflammatory signaling pathways, and modulating immune function. These results provide important insights for developing dietary intervention strategies for RA prevention and highlight the potential of optimizing antioxidant intake to reduce RA risk.

### 4.2 Mediating Role of BMI in the Relationship Between CDAI and RA

The differences between Models 3 and 4 in the results of this study suggest that the mediating role of BMI is not significant. In the mediation analysis, we found that BMI mediated approximately 10.88% of the relationship between CDAI and the risk of RA. This suggests that while BMI plays a role, the majority of the effect remains direct. Although dietary antioxidants (such as vitamin E, carotenoids, and selenium) play a significant role in reducing oxidative stress and inflammation, obesity may alter the metabolism and bioavailability of these antioxidants, thereby weakening their protective effects. This phenomenon can be explained by the following mechanisms:

**Distribution and Storage of Antioxidants**: Lipid-soluble antioxidants (e.g., vitamin E) are typically stored in adipose tissue (49). Individuals with obesity have larger amounts of adipose tissue, which may lead to a greater accumulation of antioxidants in fat tissue, reducing their availability in the bloodstream and joint tissues. This redistribution could weaken the direct role of antioxidants in mitigating oxidative stress and inflammation.
**Oxidative Stress Environment in Adipose Tissue**: Adipose tissue in individuals with obesity is often in a state of high oxidative stress, requiring more antioxidants to neutralize ROS (50). This means that even if dietary antioxidants are consumed at higher levels, their actual antioxidant effect may be offset by the obesity-induced oxidative environment.
**Dietary Fiber and Anti-inflammatory Effects**: Higher CDAI levels are generally associated with increased dietary fiber intake, which can promote gut microbiome diversity and enhance the production of anti-inflammatory short-chain fatty acids (SCFAs) (49). However, individuals with obesity often have gut microbiota imbalances (51), which may impact the metabolism and anti-inflammatory effects of dietary fiber.
**Impact of Metabolic Syndrome**: Obesity is commonly associated with metabolic syndrome, including insulin resistance, dyslipidemia, and hypertension (52). These metabolic abnormalities exacerbate the risk of RA (53) and may reduce the protective effects of dietary antioxidants.

### 4.3 Possibility of reverse causality between BMI and RA

RA itself may influence BMI, particularly due to the chronic inflammation associated with RA, which leads to weight loss, muscle mass depletion, and altered metabolic processes. While we acknowledge the possibility of reverse causality between BMI and RA, we believe BMI still plays a relevant mediating role in this study for the following key reasons.

Obesity and RA risk: Epidemiological studies consistently show that obesity is an important risk factor for the development of RA. Adipose tissue releases pro-inflammatory cytokines (such as IL-6 and TNF-α) (17), contributing to systemic inflammation that may exacerbate RA pathogenesis. This relationship suggests that BMI may increase the risk of RA, making it reasonable to consider BMI as a mediator in the relationship between CDAI and RA risk.

Chronic inflammation and obesity: The link between obesity and inflammation is well-documented in the literature, and it is increasingly recognized as a key factor in the development of various chronic diseases, including RA (54, 55). Given that dietary antioxidants have been shown to counteract inflammation, it is plausible to assume that higher antioxidant intake may mitigate obesity-related inflammation, thereby reducing RA risk.

Cross-sectional nature of the study: While we acknowledge the possibility of reverse causality, it is important to highlight that the cross-sectional design of this study provides a snapshot of the relationship between CDAI, BMI, and RA at a specific point in time. Longitudinal studies are necessary to fully explore and clarify the directionality of these relationships.

### 4.4 Clinical and public health implications

This finding provides new insights for RA prevention and management strategies. Based on the results of this study, increasing the intake of dietary antioxidants, particularly vitamin E, carotenoids, and selenium, may serve as an effective strategy for RA prevention. However, considering the mediating role of BMI, personalized dietary interventions should take participants’ weight status into account. For overweight and obese individuals, merely increasing antioxidant intake may not be sufficient to achieve the desired effects. In these populations, combining weight management strategies with increased physical activity and other comprehensive interventions may be more effective.

### 4.5 Limitations and future directions

Although this study reveals the negative correlation between CDAI and RA and the mediating role of BMI, as a cross-sectional study, the study can only establish an association between CDAI and RA and cannot infer causality. Additionally, the dietary data were obtained through 24-hour dietary recalls, which may be subject to recall bias and reporting errors. Furthermore, BMI, as a measure of obesity, does not differentiate between muscle mass and fat mass, limiting its explanatory power. In addition, the results of the present study showed that the statistical significance of CDAI in RA (p = 0.033) was relatively weak but equally statistically significant. Future studies should adopt longitudinal studies or randomized controlled trials to confirm the causal relationship between CDAI and RA risk. Moreover, further research is needed to explore the specific mechanisms of different antioxidants in RA prevention and treatment, and to evaluate the combined effects of weight management and dietary interventions.

## 5 Conclusion

This study demonstrates a significant negative correlation between the Composite Dietary Antioxidant Index (CDAI) and the risk of rheumatoid arthritis (RA) in American adults, with higher CDAI scores being associated with a reduced risk of RA. The findings highlight the potential protective role of dietary antioxidants, such as vitamin E, carotenoids, and selenium, in mitigating the inflammatory and oxidative stress components of RA. Additionally, BMI was found to partially mediate the relationship between CDAI and RA, suggesting that obesity may influence the effectiveness of dietary antioxidants in RA prevention. These results provide valuable insights into the potential of dietary interventions to reduce RA risk, particularly in high-risk populations such as those with obesity. Personalized dietary strategies that optimize antioxidant intake while considering weight management could offer a promising approach to RA prevention and management. Future longitudinal studies are necessary to confirm these findings and further elucidate the underlying mechanisms of dietary antioxidants in RA pathogenesis and treatment.

## Data Availability

The datasets presented in this study can be found in online repositories. The names of the repository/repositories and accession number(s) can be found below: All data are available in the NHANES database (www.cdc.gov/nchs/nhanes).
